# Identification and validation of methylation-CpG prognostic signature for prognosis of hepatocellular carcinoma

**DOI:** 10.18632/aging.205454

**Published:** 2024-01-18

**Authors:** Chunmei He, Zehao Guo, Hao Zhang, Ganqing Yang, Jintao Gao, Zhijing Mo

**Affiliations:** 1 School of Intelligent Medicine and Biotechnology, Guilin Medical University, Guilin 541199, Guangxi, China; 2 Key Laboratory of Biochemistry and Molecular Biology (Guilin Medical University), Education Department of Guangxi Zhuang Autonomous Region, Guilin 541199, Guangxi, China; 3 Chandi Precision Medical Technology, Foshan 528000, Guangdong, China

**Keywords:** hepatocellular carcinoma, prognostic, methylation, oxidative stress, immune checkpoint

## Abstract

Epigenetic biomarkers help predict the prognosis of cancer patients and evaluating the clinical outcome of immunization therapy. In this study, we present a personalized gene methylation-CpG signature to enhance the accuracy of survival prediction for individuals with hepatocellular carcinoma (HCC). Utilizing RNA sequencing and methylation datasets from GEO as well as TCGA, we conducted single sample GSEA (ssGSEA), WGCNA, as well as Cox regression. Through these analyses, we identified 175 oxidative stress and immune-related genes along with 4 CpG loci that are associated with the prognosis of HCC. Subsequently, we constructed a prognostic signature for HCC utilizing these 4 CpG sites, referred to as the HCC Prognostic Signature of Methylation-CpG sites (HPSM). Further investigation revealed an enrichment of immune-related signal pathways in the HPSM-low group, which demonstrated a positive correlation with better survival among HCC patients. Moreover, the methylation of the CpG sites in HPSM was found to be closely linked to drug sensitivity. *In vitro* experiments tentatively confirmed that promoter methylation regulated the expression of BMPER, one of the CpG sites within HPSM. The expression of BMPER was significantly correlated with cell death in the oxidative stress pathway, and overexpression of BMPER effectively inhibited HCC cell proliferation. Consequently, our findings suggest that HPSM is an independent predictive factor and holds promise for accurately predicting the prognosis of HCC patients.

## INTRODUCTION

Hepatocellular carcinoma (HCC) is a common cancer with high recurrence and mortality rates [1]. Increasing research has demonstrated that oxidative stress exerts a pivotal effect in the advancement of HCC [2, 3]. Additionally, abnormal gene functioning resulting from DNA methylation has been linked to cancer initiation, progression, and drug resistance [4–6]. Many studies have reported that oxidative stress can lead to abnormal hypermethylation and inactivation of tumor suppressor genes, contributing to carcinogenesis [7–9]. Therefore, methylation and oxidation-related markers may have potential effects on HCC occurrence and development. However, these markers lack perfection when predicting treatment efficacy.

The methylation of DNA is a pivotal epigenetic inheritance adorn that influences gene expression and chromosomal stability, thus contributing to tumor genesis and development. Current research on liver cancer methylation primarily focuses on differences in whole genome methylation of DNA profiles and the application of methylation markers in liver cancer detection. Targeted molecular therapy is currently a prominent area of investigation in liver cancer research [10, 11]. Recent studies have revealed that oncogene-induced oxidative stress is a key driver of CpG island hypermethylation [9, 12]. Identifying methylation markers that predict immune response and prognosis will facilitate personalized immunotherapy for HCC patients [13, 14]. Despite extensive investigation into immune-related molecular functions and interactions, there is limited research on their epigenetic regulation [15]. Gaining insight into the regulation and control of immune checkpoint genes is crucial for developing mechanistically-driven biomarkers to predict immunotherapy response. In this exploration, we employed WGCNA, single-sample GSEA (ssGSEA) analysis, as well as Cox regression to identify 175 oxidative stress and immune-related genes and 4 CpG sites associated with HCC prognosis. With these four CpG sites, we constructed the HCC Prognostic Signature of Methylation-CpG sites (HPSM), and analyzed the molecular and immune characteristics discrepancies between the HPSM-low and HPSM-high groups. Furthermore, we performed drug sensitivity analysis on the CpG sites of HPSM. Additionally, we confirmed *in vitro* the relationship between methylation of one of the CpG sites within the BMPER gene contained in HPSM and its biological function in HCC gene expression. Constructing HPSM for HCC patients may uncover underlying mechanisms between methylation and oxidative stress and the prognosis of individuals affected by HCC.

## MATERIALS AND METHODS

### Data capture

RNA-seq data from 424 HCC specimens, including 374 cancerous and 50 adjacent normal tissue specimens, along with clinicopathological information, single nucleotide variations, and 450K methylation data, were obtained from The Cancer Genome Atlas (TCGA) (http://www.cbioportal.org/). Additionally, the GSE52018, GSE14520, and GSE57956 datasets were retrieved from GEO database (https://www.ncbi.nlm.nih.gov/geo/). Patients with incomplete pathological data were excluded. The drug sensitivity file “compound activity: DTP NCI-60” and the drug methylation matrix file Illumina 450K methylation were downloaded from the Cell Miner datasets (https://discover.nci.nih.gov/cellminer).

### Identification of oxidative stress and immune related genes and prognosis-related CpG sites

Differentially expressed genes (DEGs) as well as differentially methylated genes (DMGs) among the adjacent normal tissues as well as HCC tissues in the GSE14520 and GSE57956 datasets were analyzed using GEO2R. TCGA-LIHC mRNA expression data was also analyzed using R's edge packet analysis, and DEGs were identified based on a threshold of p < 0.05 and | log 2 fold change (FC) | > 1 with statistical significance. In total, 1203 overlapping genes were got from DEGs along with DMGs. The correlation of these genes with immune and oxidative stress pathways was determined using the ssGSEA package [16] in R. After conducting GO enrichment analysis, 175 oxidative stress and immune-related genes were selected based on a correlation coefficient > |3| and p-value < 0.05. Next, univariate Cox regression analysis was carried out to explore the link of the methylation of CpG sites within these genes and overall survival (OS), filtering out prognosis-related CpG sites using a p-value threshold of less than 0.05.

### WGCNA

Co-expression networks were constructed using the “WGCNA” package in R with the overlapping genes identified from the DEGs and DMGs. Outliers were removed, and a similarity expression matrix was established by calculating the Pearson correlation coefficient between gene pairs. A soft threshold power of β = 4 was utilized to construct an adjacency matrix. The topological overlap matrix was obtained by calculating TOM values between pairwise genes. Hierarchical clustering was performed to define modules, and module-trait association analysis was established between cancer and normal phenotypes.

### Construction of HPSMs

Data normalization and conversion of DNA methylation from Beta-values to M-values were carried out utilizing the minfi package in R 4.2.2 [17]. A total of 371 HCC patients from TCGA were stochastically allocated into a training set (N=186) as well as a testing dataset (N=185). Single-variable Cox regression analysis was utilized for the screening of prognosis-related CpG sites, considering those with p < 0.05 as having prognostic value. To analyze the combined effect of multiple factors, multivariate Cox regression analysis was carried out using the LASSO method to account for multicollinearity. This step further screened candidate CpG sites. Ultimately, four CpG sites were selected as prognostic predictors for HCC, and the HPSM was constructed. A risk scoring formula was developed: Risk score = (M-value of CpG1) × coef (CpG1) + (M-value of CpG2) × coef (CpG2) + … + (M-value of CpGn) × coef (CpGn). Individuals with HCC were allocated into HPSM-high / HPSM-low groups using the median hazard threshold. Different HPSM subgroups’ OS was evaluated utilizing the Kaplan-Meier method as well as log-rank test. The performance of HPSM was evaluated by applying it to the testing dataset, generating a rROC curve, and calculating the AUC.

### Gene set enrichment analysis (GSEA) and immune characteristics analysis of HPSM subgroups

GSEA software and the clusterProfiler package in R were used to identify gene sets enriched in the HPSM-high/HPSM-low groups. The gene sets c2.all.v2023.1.Hs. symbols, immunesigdb. v2023.1.Hs. symbols and c5.go.v7.4, symbols were utilized. A false discovery rate (FDR) q < 0.05 was indicated to be statistically significant. The tumor-infiltrating immune cell subsets, immune-related functions, and tumor mutation burden (TMB) between the HPSM subgroups were estimated using the ssGSEA package [18] in R 4.2.2 and the maftools package in R 4.2.2.

### Independent prognostic analysis and generation of a nomogram

Univariate and multivariate Cox regression analyses were operated utilizing the survival package in R 4.2.2 to evaluate HPSMs’ prognostic value. Clinical characteristics such as age, gender, T, N, M, PD-1, TP53 and TMB were included as clinical-pathological covariates [19–21]. A nomogram incorporating T, M, programmed cell death 1 (PDCD1), and HPSM was constructed to forecast OS at 1, 2, and 3 years. The predictive capability together with the accuracy of the nomogram were evaluated using a ROC curve.

### Cell culture methods and 5-aza-2’-deoxycytidine treatment strategy

PLC/PRF/5 and Hep3B cell lines were subjected to DMEM medium supplemented with 10% FBS (Gibco, USA) and cultured at the temperature of 37° C with 5% CO_2_. For the treatment of PLC/PRF/5 cells, a confirmed DNA methylation inhibitor, 5-aza-2′-deoxycytidine (5-aza-dC) at 4μM, was used [22], Hep3B cells were treated with 5μM 5-aza-dC (Omega Bio-Tek, USA). The culture medium containing 5-aza-dC was replaced daily for three consecutive days.

### Bisulfite sequencing PCR (BSP)

BSP was performed to examine the methylation level of the BMPER promoter. The BSP primer sequences used were as follows: forward primer – GTGTGTCGCTCCTTCCCAAAGGTG and reverse primer - GCCCTGGGGCCCTGGCCTCC. Genomic DNA from Hep3B and PLC/PRF/5 cells was extracted using the DNA extraction kit (Omega Bio Tek). Ten randomly selected positive clones were sequenced, and the sequencing results were visualized using SeqMan software.

### Quantitative PCR (qPCR)

Total RNA was extracted utilizing TRIzol reagent (TIANGEN, Beijing, China). qPCR was performed to detect the expression of BMPER. The qPCR was carried out using the BioRad CFX96 system and SYBR Green Chemistry (BioRad, USA). The primer sequences for BMPER and GAPDH were as follows: BMPER forward -GAGCCTTGTGTTCTACGCCAGT, BMPER reverse - TACATTTGCTTCCTTCTGGCTGA, GAPDH as an internal reference forward - CATGAGAAGTATGACAACAGCCT, and GAPDH reverse - AGTCCTTCCACGATACCAAAGT. All primer sequences were obtained from BGI (Hong Kong).

### Western blotting (WB)

WB was carried out as described previously [4–6]. Anti-BMPER and anti-β-actin antibodies were purchased from ImmunoWay (USA) and Wanleibio (Shenyang, China).

### Stable expression of BMPER using lentiviral vectors

LV-BMPER (BMPER overexpression) and LV-NC (negative control) lentiviral vectors were obtained from Genechem (GENECHEM, Shanghai, China). HCC cells were infected with the lentivirus for 72 hours, followed by treatment with puromycin (Biotopped, Beijing, China). The expression levels of BMPER were detected using WB assays.

### Cell proliferation assays

Hep3B cells were seeded in 96-well plates with 5.0 × 10^3^ cells for each well, followed by incubation for 24, 48, 72, and 96 hours. After that, the cells were treated with MTT (5 mg/ml; Sigma, Dorset, UK) for 4 hours. The measurement of optical density (OD) value of each well was performed at 490nm utilizing a microplate reader.

### Clone formation assays

Paraformaldehyde and Giemsa were purchased from China National Medicines Corporation, Ltd. (Shanghai, China) and Nanjing Jiancheng Technology Company (Nanjing, China), respectively. LV-NC or LV-BMPER cells were inoculated into a 6-well plate with 500 cells/well for 10 days. Cells were fixed with 4% paraformaldehyde for a duration of 10 minutes, and then stained with 0.1% Giemsa for a duration of 10 minutes. Cell colonies were then photographed and counted. These experiments were repeated three times independently.

### Statistical analysis

Data were presented as mean ± standard deviation. The association between HPSM groups and clinicopathological characteristics was assessed using a Chi-square test. Two-group comparisons (two-tailed) were analyzed using Student's t-test, while one-way analysis of variance (ANOVA) was utilized for comparisons involving more than two groups. Analysis of Pearson correlation was performed. The predictive efficiency of the survival risk score was evaluated using the ROC curve. Univariate and multivariate Cox regression analyses were conducted to predict the variables for the prognosis and clinicopathological characteristics. Data analyses were carried out based on GraphPad Prism 8.0 software together with R 4.0.2. *P <* 0.05 indicated statistically significant.

### Availability of data and materials

The datasets of this article were generated from the TCGA database and the GEO database.

## RESULTS

### WGCNA of DEGs and DMGs in HCC

**Figure 1:**
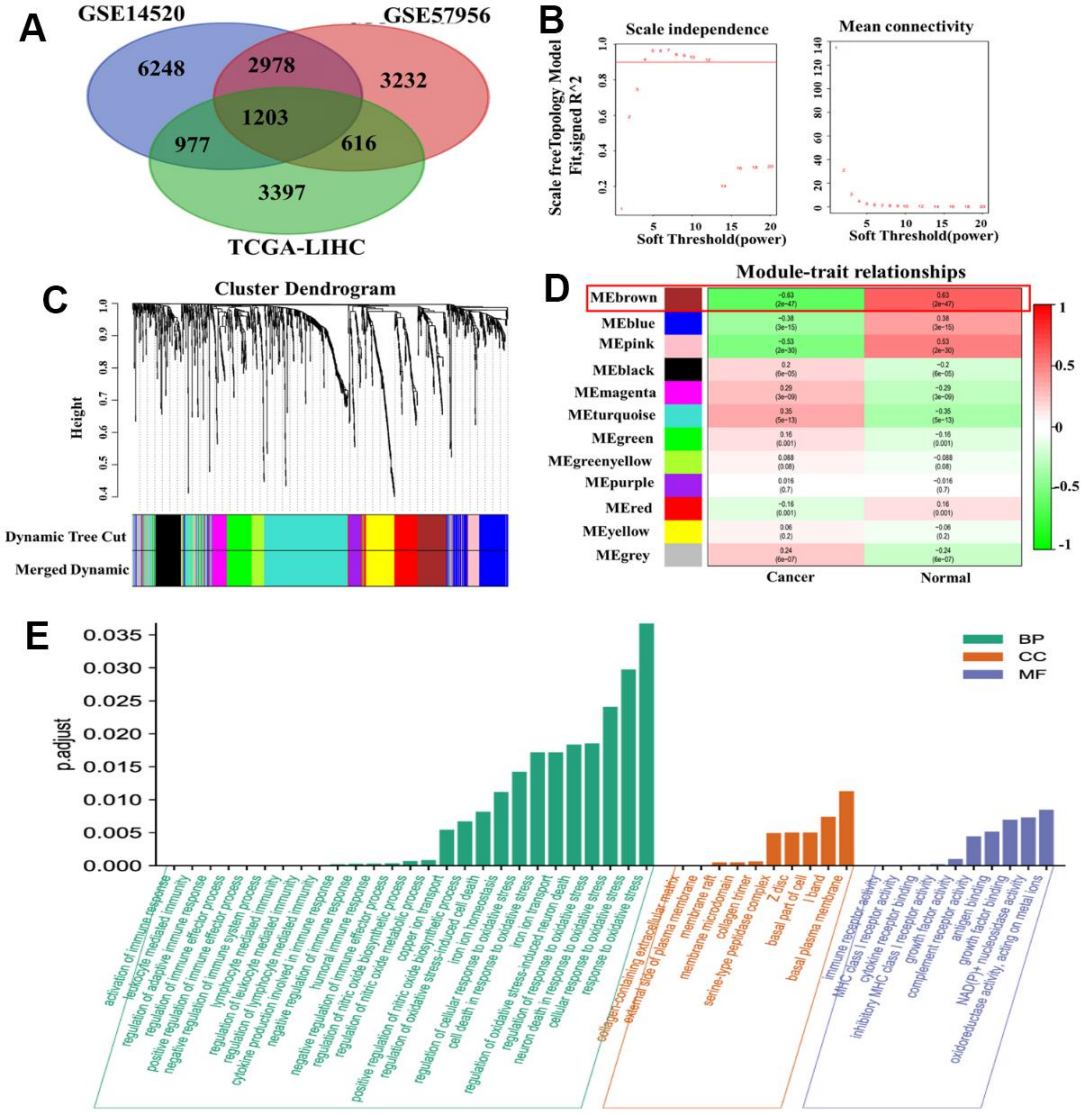
**WGCNA of DMGs and DEGs in HCC.** (**A**) Identification of overlapping genes between DMGs and DEGs. (**B**) Analysis of the scale-free fit index for different soft threshold powers (β). (**C**) Clustering dendrogram revealing the presence of 12 modules. (**D**) Correlation matrix showing the relationships between these modules in normal and cancer tissues: red indicates positive correlations, while green represents negative correlations. (**E**) GO enrichment analysis of 175 genes associated with oxidative stress and immunity.

We obtained a total of 11,045 DEGs from the GSE14520 dataset and 8,029 DMGs from the GSE57956 dataset using GEO2R. Additionally, we evaluated 6,219 DEGs in the TCGA-LIHC RNA-seq expression profile. Among these, we identified 1,203 genes that overlapped between the DMGs and DEGs related to HCC (Figure 1A). To construct a co-expression network, we chose a soft-thresholding power (β) of 4, resulting in a WGCNA containing the overlapping genes (Figure 1B). The co-expression network formed 12 modules, each consisting of at least 30 genes and represented by different colors (Figure 1C). Notably, the brown module showed a strong association with the HCC phenotype (Figure 1D). Through gene ontology (GO) enrichment analysis, we found 175 genes within the brown module that were associated with immune and oxidative stress biological processes, including modulation of cellular response to oxidative stress, neuron death in response to oxidative stress, activation of immune response, and regulation of immune effector process (Figure 1E).

### Construction of HPSM

**Figure 2:**
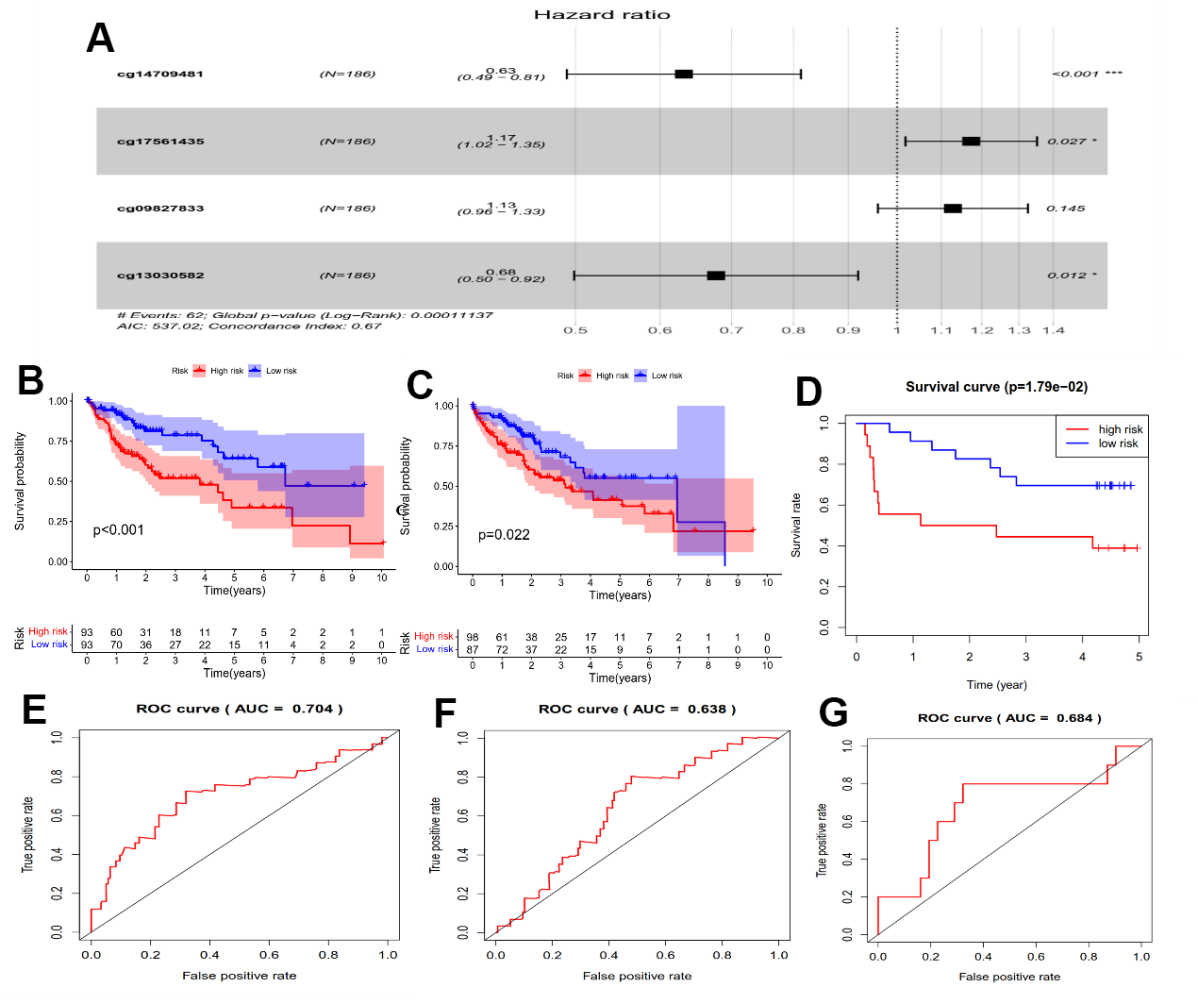
**Prognostic analysis of the HPSM subgroups.** (**A**) Forest plot displaying the hazard ratios (HRs) for 4 CpGs and OS. (**B**) Kaplan-Meier survival curves for OS comparing HPSM-high group with HPSM-low group within the TCGA training dataset. (**C**) Kaplan-Meier survival curves for OS comparing HPSM-high group with HPSM-low group within the TCGA testing dataset. (**D**) Kaplan-Meier survival curves for OS comparing HPSM-high group with HPSM-low groups within the GSE52018 dataset. (**E**) ROC curves to predict the OS between HPSM groups within the TCGA training dataset. (**F**) ROC curves for OS prediction between HPSM groups within the TCGA testing dataset. (**G**) ROC curves for OS prediction between HPSM groups within the GSE52018 dataset.

We performed Cox regression analysis on the methylation-CpG sites of the 175 genes related to oxidative stress and immunity to determine key prognostic methylation-CpG sites. The results revealed that four methylation-CpG sites significantly affected the prognosis of HCC patients: cg14709481, cg09827833, cg13030582, and cg17561435 (Figure 2A). Subsequently, a HPSM risk score was constructed using the following formula: risk score = (-0.45938 × M-value of cg14709481) + (0.120214 × M-value of cg09827833) + (0.15969 × M-value of cg17561435) + (-0.38992 × M-value of cg13030582). Utilizing the median observed risk score, the training dataset (n=186) were divided into two groups: HPSM-high (n=93) as well as HPSM-low (n=93). The HPSM-high group’s OS was significantly lower in contrast with that of the HPSM-low group (Figure 2B, *p*<0.01). We evaluated the accuracy of the HPSM prediction model using the AUC, which yielded a value of 0.704 for OS (Figure 2E). The testing dataset (n=185) were divided into HPSM-high (n=98) as well as HPSM-low (n=87) groups. Significant differences in OS rates were observed in the HPSM-high and HPSM-low groups (Figure 2C, *p*<0.05), with an AUC value of 0.638 (Figure 2F). The GSE52018 dataset was used as exterior data to validate the HPSM model’s accuracy and reliability, and the survival curve and ROC curve yielded consistent conclusions (Figure 2D, 2G).

### Molecular characteristics and immunological characterization of the HPSM subgroups

**Figure 3:**
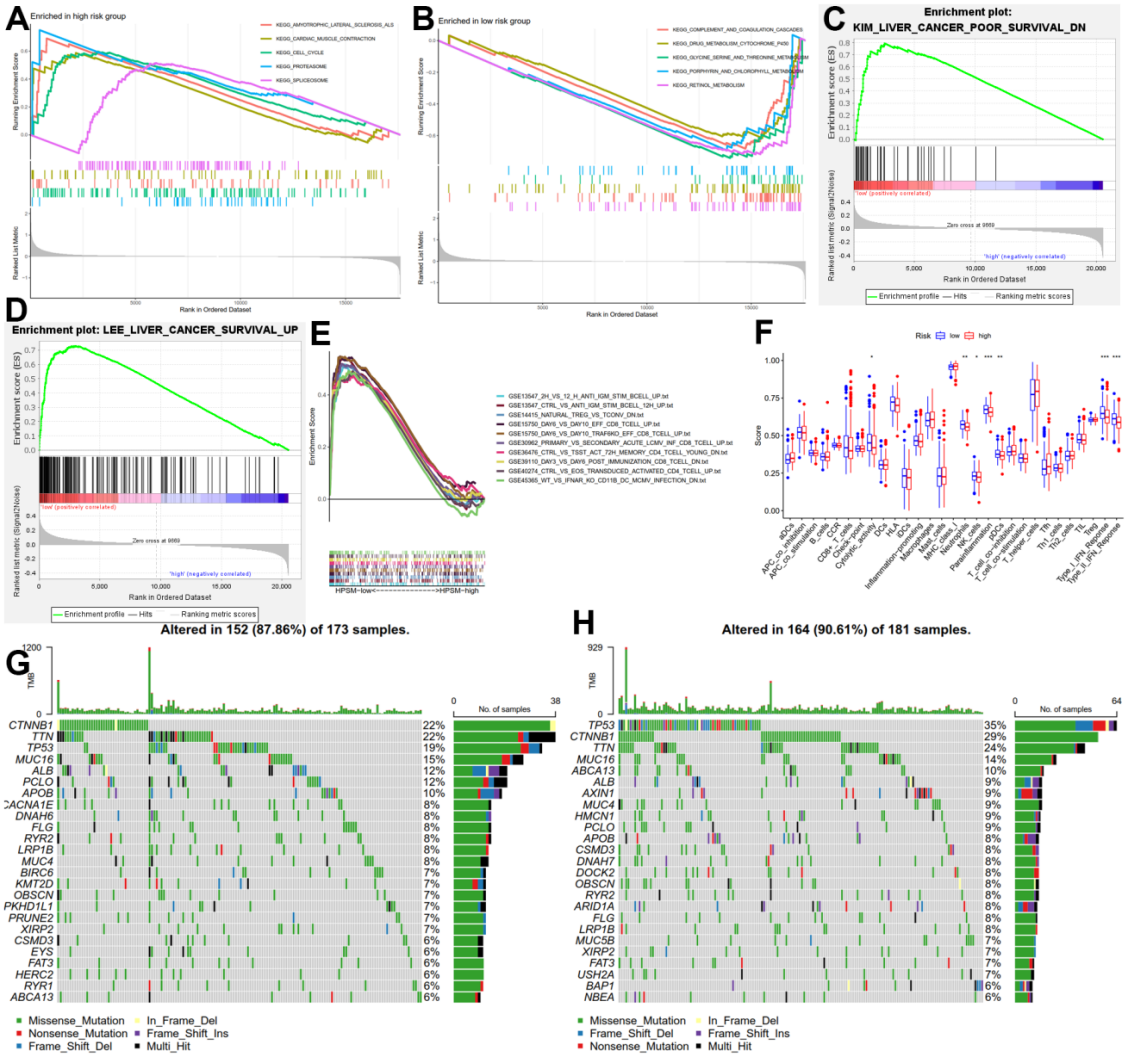
**Molecular and immune function analysis among HPSM groups.** (**A**) Enriched gene sets among the HPSM-high group. (**B**) Enriched gene sets among the HPSM-low group. (**C**, **D**) GSEA of HPSM subgroups in survival-related gene sets. (**E**) GSEA enrichment analysis of HPSM subgroups in immune-related gene sets. (**F**) Scores of 29 tumor-infiltrating immune cell subsets and immune-related functions. (**G**) TMB analysis in HPSM-low groups. (**H**) TMB analysis in HPSM-high groups.

To explore key signaling pathways in the HPSM subgroups, we performed GSEA on gene sets enriched in the HPSM-high/HPSM-low groups. Results showed that the HPSM-high group was enriched in cell cycle, peroxisome, as well as spliceosome pathways (Figure 3A). On the other hand, the HPSM-low group showed enrichment in metabolic pathways such as drug metabolism cytochrome, threonine metabolism, P450, glycine, serine, porphyrins and chlorophyll metabolism, and retinol metabolism (Figure 3B). Moreover, gene sets associated with good survival of HCC patients were enriched in the HPSM-low group (Figure 3C, 3D). By conducting GSEA focused on immune pathways, we found that 52 pathways related with immune were enriched among the HPSM-low group but not among the HPSM-high group (Figure 3E). Additionally, using ssGSEA, we observed higher levels of tumor-infiltrating immune cell subsets along with immune-associated functions among HPSM-low group in contrast with the HPSM-high group (Figure 3F). Furthermore, the 25 genes with the highest mutation rates were identified among the HPSM subgroup (Figure 3G, 3H). TP53, TTN, CTNNB1, and MUC16 exhibited mutation rates exceeding 13% in both groups. Notably, TP53 mutations showed the largest difference in mutations between the HPSM subgroups, with a higher prevalence in HPSM-high samples (35%) compared to HPSM-low samples (19%).

### Prognostic analysis of HPSM and nomogram development

**Figure 4:**
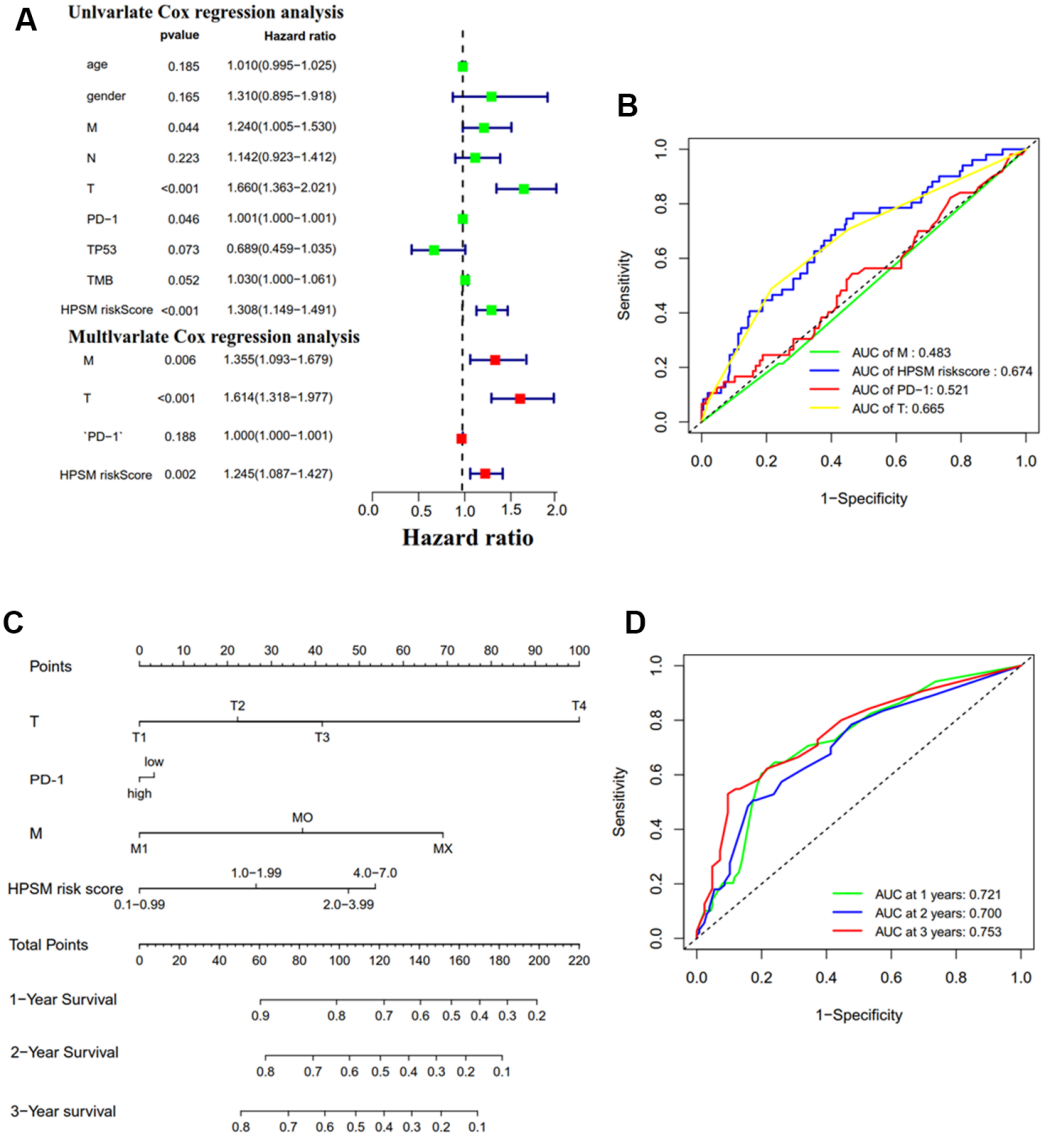
**Prognostic analysis of HPSM and nomogram development.** (**A**) Univariate and multivariate Cox regression analyses of HPSM risk score as well as prognostic parameters. (**B**) ROC curves of HPSM risk score and prognostic parameters. (**C**) Prognostic nomogram predicting 1-, 2-, and 3-year OS of HCC individuals. (**D**) ROC curves of the prognostic nomogram.

Univariate and multivariate Cox regression analyses was operated to evaluate the association between HPSM hazard scoring, M staging, T staging, and the prognosis of HCC patients (Figure 4A). ROC curve’s AUC was calculated as 0.674 for the HPSM hazard scoring (Figure 4B). Based on these results, we developed a nomogram that demonstrated a higher contribution to the model with increasing HPSM risk scores, and lower 1-, 2-, and 3-year survival rates (Figure 4C). The AUCs were found to be 0.721, 0.700, and 0.753 for 1-, 2-, and 3-year OS of HCC patients, respectively (Figure 4D). Additionally, the clinicopathological characteristics of the patients indicated significant correlations between the HPSM groups and multinodularity, Cancer of the Liver Italian Program (CLIP) staging, Alpha-fetoprotein (APF), and survival times of HCC patients (Table 1). Therefore, the HPSM risk score may act as an important prognostic indicator for HCC individuals.

**Table 1:** Relationship of HPSM risk score with clinicopathological characteristics of HCC.

**Clinicopathological parameters**	**n**	**HPSM groups**		**P**
		**HPSM-high**	**HPSM-low**	
gender				
Male	39	18	21	0.581
Female	2	0	2	
Age				
≤50	26	13	13	0.300
>50	15	5	10	
ALT				
Low	22	8	14	0.200
High	19	10	9	
Main tumor size				
≤3 cm	21	7	14	0.162
>3 cm	20	11	9	
Multinodular				
No	29	10	19	0.018
Yes	12	9	3	
Cirrhosis				
No	5	1	4	0.504
Yes	36	17	19	
TNM staging				
I	15	4	11	0.172
III+II	18	9	9	
BCLC staging				
0+A	22	8	14	0.620
B+C	11	5	6	
CLIP staging				
0+1+2	28	8	20	0.012
3+4+5	5	5	0	
AFP				
≤300 ng/ml	21	4	17	0.001
>300 ng/ml	20	14	6	
survival status				
Alive	23	7	16	0.05
Dead	18	11	7	
survival times				
≤6 months	8	8	0	0.002
>6 months	33	10	23	
recurrence status				
False	13	3	10	0.085
True	27	14	13	
recurrence times				
≤36 months	23	12	11	0.150
>36 months	17	5	12	

### Drug sensitivity analysis

**Figure 5:**
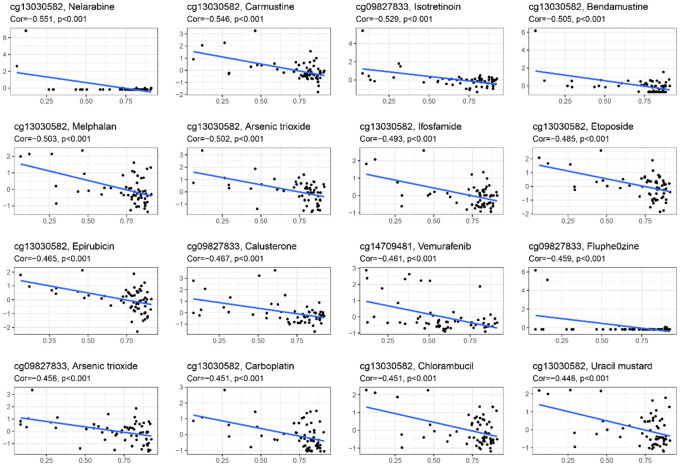
Relationship between methylation levels of CpG sites in the HPSM and FDA drug sensitivity.

A drug sensitivity experiment was conducted to study the relationship between CpG sites’ methylation levels in the HPSM and drug sensitivity. It was found that the methylation of cg13030582 was in a negative correlation with sensitivity to nelarabine, carmustine, bendamustine, melphalan, arsenic trioxide, ifosfamide, etoposide, epirubicin, carboplatin, chlorambucil, and uracil mustard. Methylation levels of cg09827833 were also negatively correlated with sensitivity to isotretinoin, calusterone, fluphenazine, and arsenic trioxide. Furthermore, the methylation level of cg14709481 showed a negative correlation with sensitivity to vemurafenib (Figure 5). These results suggest that the methylation levels of these specific CpG sites in HPSM may be associated with drug sensitivity, providing a clue for further investigation into individualized therapy for HCC patients.

### Regulation of BMPER expression by promoter methylation

**Figure 6:**
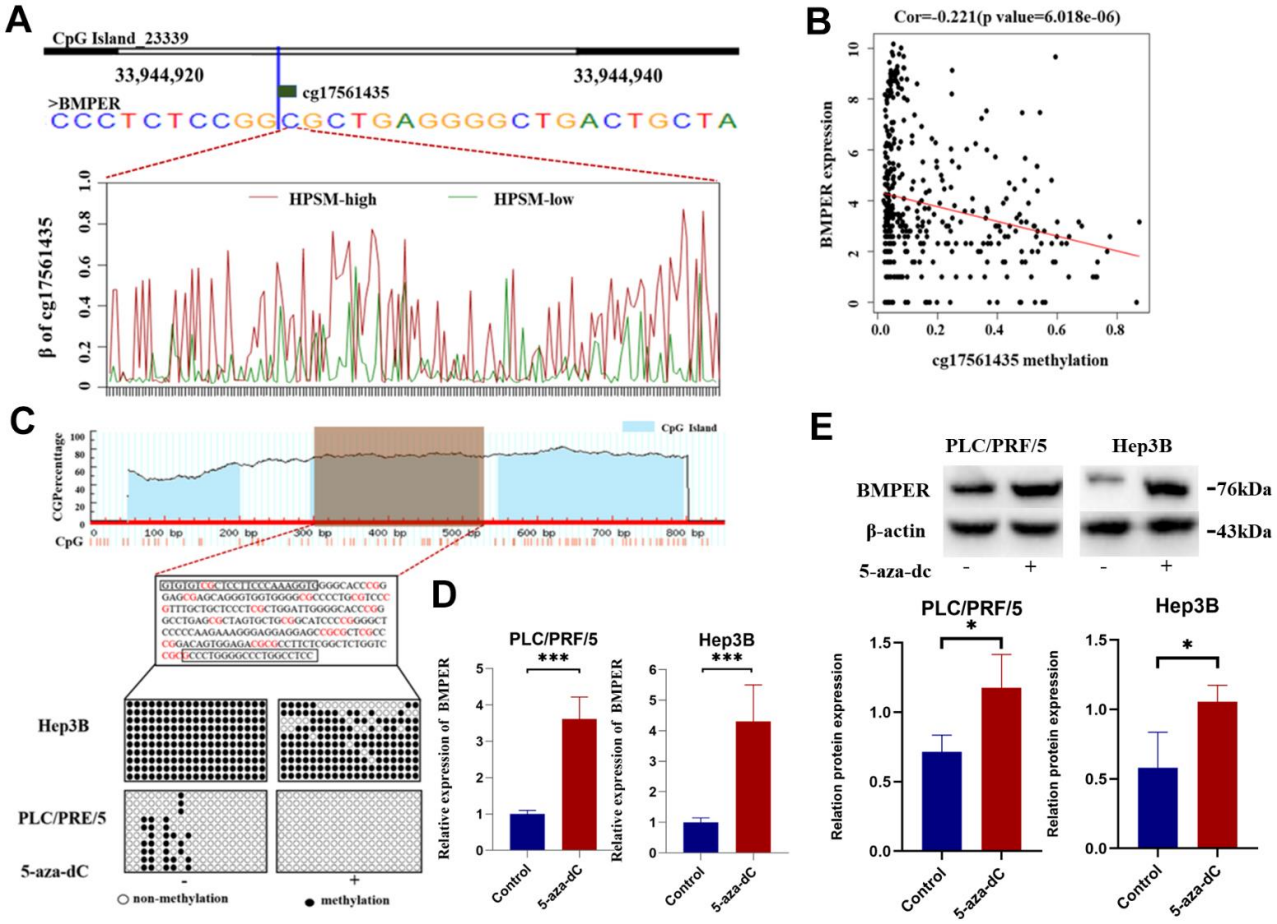
**Regulation of BMPER expression by promoter methylation.** (**A**) Methylation level of cg17561435 in different HPSM subgroups. (**B**) Negative correlation between BMPER mRNA expression and cg17561453 methylation levels. (**C**) Prediction of CpG islands in the BMPER promoter using the MethPrimer website, and detection of BMPER promoter methylation status using bisulfite sequencing PCR (BSP). (**D**) Quantification of BMPER mRNA expression levels after 5-aza-dC treatment using qPCR (***p < 0.01). (**E**) Detection of BMPER protein expression levels after 5-aza-dC treatment using western blotting.

Among the CpG sites in HPSM, cg17561435 exhibited a greater methylation level among the HPSM-high group compared to HPSM-low group (Figure 6A). The 5' end of the BMPER gene contains a CpG island spanning 1844 bp and consisting of 153 CpG sites. Methylation of the cg17561435 site was found to be negatively correlated with BMPER mRNA expression (Figure 6B). To confirm this regulation, the 19 CpG sites’ methylation level among CpG islands using BSP was analyzed. The results demonstrated a significant reduction in BMPER methylation levels after treatment with 5-aza-dC compared to controls (Figure 6C). Additionally, both qPCR and WB showed increased mRNA / protein levels of BMPER in both cell lines following 5-aza-dC therapy (*p* < 0.05) (Figure 6D, 6E). These findings indicated that BMPER expression could be regulated by promoter methylation.

### Inhibition of HCC cell proliferation by BMPER

**Figure 7:**
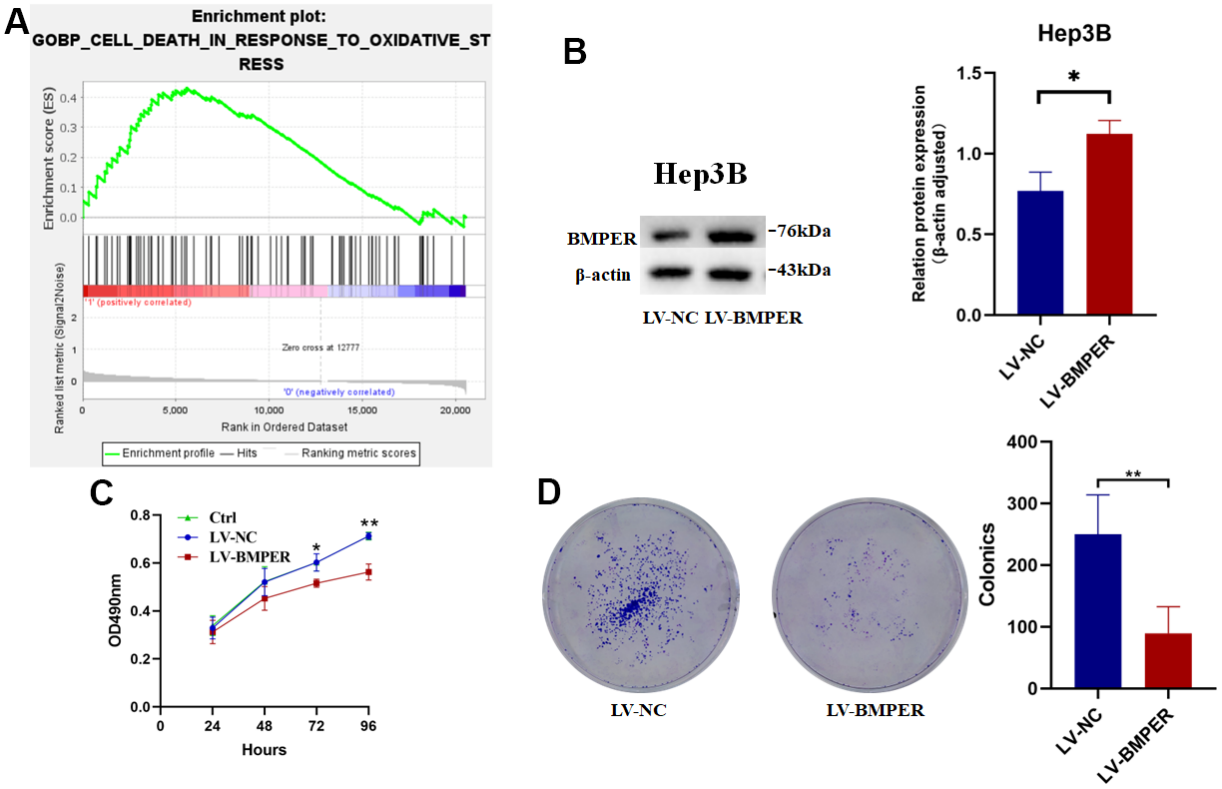
**Inhibition of HCC cell proliferation by BMPER overexpression.** (**A**) GO biological process pathway enrichment analysis using GSEA. (**B**) Confirmation of BMPER overexpression in Hep3B cell line using western blotting. (**C**) Effects of BMPER overexpression on cell proliferation assessed by MTT assays. (**D**) Clonal formation assays comparing the LV-NC and LV-BMPER groups.

To investigate BMPER’s biological function in HCC, GSEA enrichment analysis was conducted. We found that cell death in oxidative stress biological process was enriched in the group with low BMPER expression levels (Figure 7A). To further validate these results, we established a BMPER overexpression model in the Hep3B cell line (Figure 7B). As expected, MTT experiments revealed a significant reduction in cell proliferation among the LV-BMPER group in contrast with LV-NC group (*p* < 0.05) (Figure 7C). Moreover, clone formation assays demonstrated a significant decrease in clone-forming ability in the LV-BMPER group (Figure 7D). These findings confirmed that BMPER overexpression inhibited the proliferation of HCC cells.

## DISCUSSION

Algorithms that integrate DNA methylation prognostic markers, oxidative stress, and immune function indicators have shown improved prediction of markers for the prognosis as well as therapeutic targets [23, 24]. In the present study, 175 genes related to oxidative stress and immunity were identified. Subsequently, we constructed the HPSM consisting of four methylation sites: cg14709481 of HK3, cg09827833 of TEK, cg13030582 of MFAP4, and cg17561435 of BMPER. The TCGA training and testing datasets confirmed a poor prognosis among HCC patients showing a high HPSM risk score, and this finding was also validated in external GEO cohorts. HK3, an isomer of hexokinase responsible for the initial step of glucose metabolism and cell protection against oxidant-induced death, particularly in immune cells [25–27]. Studies have shown that HK3 is associated with immune infiltration; besides it can predict the response to immunotherapy [28]. In our HPSM model, the hypomethylation of cg14709481 in HK3 was significantly associated with a higher HPSM risk score, suggesting that cg14709481 hypomethylation may serve as a poor prognostic factor for HCC patients. TEK, a receptor tyrosine kinase expressed in vascular endothelial cells, exerts its effect on endoplasmic reticulum stress-induced cell death [29]. Monocytes expressing TIE2 not only regulate HCC angiogenesis but also suppress the activation of T cells and promote the expansion of regulatory T cells [30–32]. Consistent with our findings, MFAP4 has been proposed as a molecular marker for HCC diagnosis and prognosis [33]. BMPER binds to BMP and regulates TGF-β/BMP signaling. Previous studies have linked BMPER to lung cancer and ovarian cancer [34, 35], and it has been shown that BMPER expression is controlled by methylation at the transcriptional level. Treatment with 5-aza-dC can reduce BMPER expression in fibroblasts and mitigate invasion and migration of idiopathic pulmonary fibrosis lung fibroblasts [35]. In our study, we observed a positive correlation between hypermethylation of cg17561435 in BMPER and the HPSM risk score, suggesting that BMPER hypermethylation may be closely associated with poor prognosis in HCC.

Clinical trials have demonstrated that the absence of clinical benefits from cell cycle passage correlates primarily with TP53 mutations [36]. TP53 mutation is associated with more invasive diseases and poorer outcomes [37, 38] in cancer patients, particularly those with HCC [39, 40], TP53 can affect the cell cycle pathway through p53/TGF-β signaling [41]. Interestingly, we found enrichment of the cell cycle pathway in the HPSM-high group, where TP53 mutations were more prevalent and the prognosis was worse compared to the HPSM-low group. These results suggest that TP53 mutations may influence the cell cycle pathway and contribute to a poorer prognosis in the HPSM-high group.

Two gene sets related to good prognosis in HCC patients were enriched in the HPSM-low group, indicating better OS for HPSM-low group in contrast with HPSM-high group, consistent with prognostic prediction on the basis of the HPSM risk score. Higher levels of tumor-infiltrating immune cells have been demonstrated to be generally linked to a better prognosis [42–44]. Neutrophils, natural killer (NK) cells, plasma cytotoxic dendritic cells (pCDs), and type I and II interferon (IFN) reactions were obviously higher among the HPSM-low group in contrast with HPSM-high group. Studies have shown that type II immune interferons (IFNs) are in a close association with favorable clinical outcomes within various cancer types [45–47]. which is consistent with our findings. Furthermore, we observed enrichment of the amino acid metabolism pathway among the HPSM-low group. A recent study reported that amino acid metabolism enhances the immune response against tumors [48]. Serine, in particular, is an essential nutrient for T cell responses and a critical mediator of the anti-tumor immune response [49–51]. Taken together, our results suggest that the HPSM-low group elicits a more robust tumor immune response than the HPSM-high group, leading to better OS.

Cox regression analyses revealed that HPSM risk scoring, M staging, and T staging are independent prognostic factors for HCC. HPSM risk score’s AUC (0.674) was higher compared to that of T stage (0.665) and M stage (0.483), indicating that the HPSM risk score is a more accurate prognostic factor. Thus, we developed a prognostic nomogram model by integrating traditional clinical features with the HPSM risk score. This score significantly contributes to the predictive ability of the entire nomogram, showing robust performance (AUC≥0.7). Our study suggests that the HPSM risk score is a valuable independent prognostic indicator. Patients with multinodular HCC accompanied by vascular invasion and microvascular invasion have more invasive tumors and a higher rate of recurrence, leading to poor prognosis [52]. The CLIP staging system provides more accurate prognostic information than the Child-Pugh and Okuda classifications, with higher scores indicating worse prognosis in HCC patients [53–55]. Numerous studies have established a strong association between higher levels of AFP in the blood and worse prognosis and increased risk of recurrence in HCC patients [56–58]. In this study, we found that the HPSM risk score correlates with these clinicopathological characteristics and the survival time of HCC patients, highlighting its significant role in the course of HCC. The HPSM risk score may be an important biomarker for estimating the risk of HCC recurrence, progression, further facilitating the selection of treatment options.

The CpG sites of HPSM are closely related to FDA-approved drugs. Previous studies have demonstrated that isotretinoin, fluphenazine, and arsenic trioxide induce oxidative stress as part of disease treatment [59–62]. Interestingly, the methylation level of cg09827833is negatively correlated with isotretinoin, arsenic trioxide, and fluphenazine. Methylation of cg14709481 is negatively associated with the sensitivity of the BRAF inhibitor vemurafenib. Several studies have highlighted the immunomodulatory effects of anti-tumor drugs [63]. BRAF inhibitors, for example, increase levels of immunostimulatory cytokines, reduce immunosuppressive cytokines, and decrease T cell infiltration and activity in tumors through interference with the MAPK signaling pathway [64]. Combining BRAF inhibitors with immune checkpoint inhibitors may lead to better tumor suppression. The methylation level of cg13030582 is negatively correlated with immunomodulatory inhibitors such as isotretinoin, DNA synthesis inhibitors (etoposide, epirubicin, carbolatin), and DNA alkylating agent carmustine. Isotretinoin induces apoptosis of liver cancer cells by reducing the activities of superoxide dismutase (SOD), peroxidase (POD), and glutathione (G-SH) [65]. Etoposide mediates reactive oxygen species (ROS) production and induces necrosis in HK-2 cells through a p53-mediated anti-apoptotic pathway [66]. Carbolatin promotes LSCC cell apoptosis through the induction of oxidative stress and ROS production [67]. Carmustine induces ROS production and promotes neurotoxicity [68]. The combination of tumor immunotherapy and oxidative stress represents a potentially effective strategy for tumor treatment, enhancing antitumor activity mediated by immune cells and inducing oxidative stress in tumor cells. HPSM may serve as a potential target for oxidative stress and immunomodulators or an effective marker for predicting drug sensitivity.

In our study, the methylation level of cg17561435 was demonstrated to be positively linked to the HPSM risk score. Furthermore, we confirmed that BMPER expression was associated with cell death in response to oxidative stress, and BMPER overexpression inhibited the proliferation of HCC cells. Whether the role of BMPER in inhibiting tumor cell proliferation is caused by oxidative stress needs further verification. These results provided evidence for the crucial function of BMPER in HCC progression, necessitating further investigation.

## CONCLUSIONS

In conclusion, the HPSM, constructed based on four CpG sites, serves as a valuable independent prognostic factor. Additionally, the HPSM risk score could predict the efficacy of immunotherapy in HCC patients. Moreover, the CpG sites were associated with drug sensitivity, providing guidance for individualized treatment approaches for HCC.
